# Vector-borne nematode diseases in pets and humans in the Mediterranean Basin: An update

**DOI:** 10.14202/vetworld.2019.1630-1643

**Published:** 2019-10-26

**Authors:** Djamel Tahir, Bernard Davoust, Philippe Parola

**Affiliations:** 1 Aix Marseille Univ, IRD, AP-HM, SSA, VITROME, Marseille, France; 2 Institut Hospitalo-Universitaire Méditerranée Infection, Marseille, France; 3 Microbes Evolution Phylogeny and Infections, Aix Marseille Univ, Institut de Recherche pour le Développement, Assistance Publique–Hôpitaux de Marseille, Institut Hospitalo-Universitaire Méditerranée Infection, Marseille, France

**Keywords:** arthropods, companion animals, humans, Mediterranean Basin, one health, parasites, vectors, zoonoses

## Abstract

Vector-borne diseases (VBDs) are among the leading causes of morbidity and mortality in humans and animals. The scale of VBDs is increasing worldwide, including in the Mediterranean Basin, a region exposed to climate changes. Indeed, weather conditions may influence the abundance and distribution of vectors. The vector-borne nematode diseases of dogs and cats, such as dirofilariosis, onchocercosis, thelaziosis, *Cercopithifilaria*, and *Acanthocheilonema* infections, are some of these vectorized diseases, several of which are zoonoses. They are all caused by parasitic nematodes transmitted by arthropods, including mosquitoes (*Dirofilaria* spp.), black flies (*Onchocerca* lupi), drosophilids (*Thelazia* callipaeda), ticks (*Acanthocheilonema dracunculoides* and *Cercopithifilaria bainae*), and fleas and lice (*Acanthocheilonema reconditum*). The control and prevention of these infections and diseases require a multidisciplinary approach based on strengthening collaboration between the different actors in the fields of health, research, sociology, economics, governments and citizens, to improve human, animal, and ecosystem health. This is the concept of “one health.” The review aimed to provide a general update on the spatial and temporal distribution of vector-borne nematodes diseases affecting companion animals and humans, as well as the vectors involved in the Mediterranean area. Simultaneously, certain epidemiological parameters, diagnosis, treatment, and control of these diseases based on the “one health” concept will also be discussed.

## Introduction

Vector-borne diseases (VBDs) are caused by viruses, bacteria, or parasites transmitted from one infected and infectious vertebrate host to another through the bite of bloodsucking arthropods (ticks, fleas, lice, mosquitoes, sand flies, etc.) during blood meals. These diseases represent more than 17% of all known infectious diseases, causing more than 1 million deaths annually [1]. Approximately 60% of the emerging infectious diseases are zoonotic [2]. VBDs are commonly found in tropical and subtropical regions, especially in developing countries [1]. This is particularly true for some of the most important diseases transmissible to humans (such as malaria, trypanosomosis, Chagas disease, and lymphatic filariasis) and animals (such as theileriosis, babesiosis, and trypanosomosis). The distribution of these diseases appears to be worldwide regarding companion animals (dogs and cats). This is the case, for example, for dirofilariosis, leishmaniosis, ehrlichiosis, anaplasmosis, and cytauxzoonosis [3].

**Figure-1:**
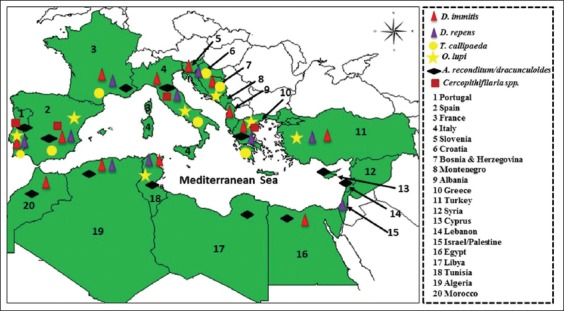
Geographic distribution of vector-borne helminths in the Mediterranean Basin. Distribution of *Dirofilaria immitis*, *Dirofilaria repens*, *Thelazia callipaeda*, *Onchocerca lupi*, *Acanthocheilonema reconditum*/*dracunculoides*, and *Cercopithifilaria* spp. detected in companion animals and/or in humans.

Vector-borne nematodes belong to the order *Spirurida*, suborder Spirurina and families Filariidae and Onchocercidae [4]. They are prevalent in the Mediterranean Basin (Figure-1) and some of them are of growing medical and veterinary importance [3,5]. In fact, the Mediterranean climate is favorable to the stable development of many arthropod species which are implicated as vectors for different agents, including helminths [6]. Accordingly, humans living in these areas, as well as their domestic and companion animals, are potentially exposed to the risk of disease-causing vector-borne nematodes [3,7,8]. Of the vector-borne nematode diseases (VBNDs), dirofilarioses are the most important, involving two species, *Dirofilaria immitis* and *Dirofilaria repens*, and affecting domestic and wild canids, causing cardiopulmonary (also known as heartworm disease) and subcutaneous dirofilariosis, respectively [9,10]. In humans, *D. immitis* and *D. repens* cause pulmonary and subcutaneous/ocular dirofilariosis, respectively [10]. At the same time, in several regions of world (including Southern Europe), thelaziosis represents an emerging VBND in which the nematode *Thelazia callipaeda* (known as “oriental eye worm”) has been reported as a causal agent for animal (dogs, cats, and foxes) and human ocular thelaziosis [3]. Another species of *Thelazia*, *Thelazia californiensis* (the “Californian eye worm”) may also infect humans and dogs. However, this is limited to Western areas of the United States of America. It should be noted that all species of *Thelazia* are transmitted by non-biting insect vectors, which feed on eye secretions [11]. In terms of canine ocular onchocerciasis, *Onchocerca lupi*, originally described in a wolf, has been recognized as the causative agent. This has been reported worldwide, particularly in the United States and Europe [12]. Its zoonotic role was confirmed after the first signs of human infection with *O. lupi* in Turkey in 2011 [12]. *Acanthocheilonema dracunculoides* and *Acanthocheilonema reconditum* are the causative agents of *Acanthocheilonema* infection of dogs in which adult parasites live in the peritoneal cavity often discovered accidentally during intra-abdominal surgical procedures or in the subcutaneous tissues causing subcutaneous nodule formations. These two species have a different geographical distribution: *A. reconditum* has been reported in Europe, Asia, and Africa, while *A. dracunculoides* has a more widespread distribution [13]. So far, no human cases officially caused by *A. dracunculoides* have been reported, but their zoonotic potential should not be neglected [14]. Moreover, cases due to *A. reconditum* have been described in Australia in the human eyes [14]. Other filarioids, such as *Cercopithifilaria* spp., have been incriminated in parasitic diseases of dogs in certain regions of the world [15,16]. In the past few years, *Cercopithifilaria* spp. have widely been reported in dogs from Europe and the Mediterranean region, although this has often been neglected [7]. It should be noted that the pathology has not yet been described in humans [17].

In the countries of the Mediterranean region, VBND of dogs and cats is attracting attention because of the risk of spread to previously non-endemic areas and because most of them are emerging zoonotic agents. In Europe, particularly in countries bordering the Mediterranean Basin, the epidemiology (occurrence, risk, transmission, etc.) of VBND of pets has been widely investigated (especially by Otranto *et al*. [3]), and data about these infections and diseases have been published [3]. However, on the southern shore of the Mediterranean, the data seem fairly localized and are not up-to-date. In this review, the authors present an overview of the various VBNDs of pets and humans described to date in the Mediterranean Basin. They also discuss the epidemiology, diagnosis, treatment, and surveillance of these VBNDs.

## Dirofilariosis

### Etiology

Dirofilarioses are mosquito-borne parasitic diseases, mainly of dogs and wild canids, caused by nematodes of the genus *Dirofilaria* (*Spirurida*: *Onchocercidae*). *D*. *immitis* and *D. repens* are the best-known filarioids affecting dogs and are the most frequently found species in the world [18]. Both parasites are zoonotic, but *D. repens* seems to have a high zoonotic potential in comparison with *D. immitis* [19], and they may cause serious infections in humans, with three clinical forms: Pulmonary (*D. immitis*), subcutaneous, and ocular (*D. repens*) dirofilariosis [10,20]. Nevertheless, this specificity species/clinical form is often not the case, since recent molecular studies have reported that human subcutaneous and cutaneous-pulmonary dirofilariosis were due to *D. immitis* and *D. repens*, respectively [21,22].

### Host and life cycle

**Figure-2:**
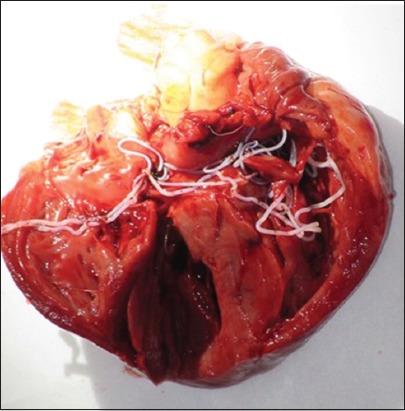
Adult worms of *Dirofilaria immitis* in the heart of a dog.

**Figure-3:**
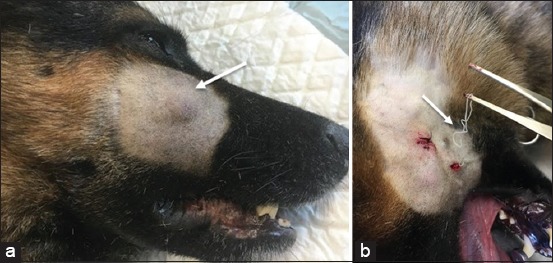
Canine subcutaneous dirofilariasis caused by *Dirofilaria repens*, (a) Subcutaneous nodule in the cheek of a male dog, (b) Adult worm after surgically opened subcutaneous nodule.

The natural transmission of *Dirofilaria* species between animals, dogs, and cats and from animals to humans usually occurs through a mosquito bite. During a blood meal, an infected mosquito introduces third-stage filarial larvae (L3) of *D. immitis* or *D. reens* into the skin of the definitive host, which is usually a domestic dog [10]. In general, the prepatent period is 120-180 days for *D. immitis* and 189-259 days for *D. repens* [10]. For *D. immitis*, adults reside in the pulmonary arteries and are occasionally found in the right ventricle of the heart (Figure-2). Adult females are usually 230-310 mm long by 350 µm wide; males are usually 120-190 mm long by 300 µm wide. Concerning *D. repens*, adult worms reside in the subcutaneous tissues (Figure-3). Adult females are usually 100-170 mm long by 460-650 µm wide; males are usually 50-70 mm long by 370-450 µm wide. Over their lifespan, females release microfilariae into the bloodstream of the definitive host and can be observed on a blood smear [23]. Microfilariae are ingested by mosquitoes during their blood meal and develop through the mosquito vectors from first-stage larvae to third-stage infective L3 larvae in 10-14 days [10].

### Epidemiology

The dirofilarioses are present in several parts of the world and have a heterogeneous distribution. *D. immitis* is more prevalent in tropical and temperate regions, particularly in the Southeast of the United States of America, in many countries of South America, in Australia, in Asia, and Southern Europe. Meanwhile, *D. repens* appears to be exclusive to the Old World [24,25].

The Mediterranean region is characterized by the coexistence of both *D. immitis* and *D. repens* species [25,26]. In this area, canine and human dirofilarioses are endemic [27] and are detected with increasing frequency in Mediterranean countries [28]. Nevertheless, the number of reported cases varies between regions, particularly when comparing the northern (high prevalence) and southern (low prevalence) shores of the Mediterranean. For example, infections with *D. immitis* in dogs range from 5% to 80% in the Northern regions of Italy [29]. In this country, the Po River Valley and areas to the north have long been considered as the main focus for heartworm infection [24]. In Spain, prevalence ranges from 8% to 36% [30]. The Province of Salamanca (in west-central Spain) and the Iberian Peninsula have been considered endemic areas, although there has been a significant decrease in prevalence (5.8% in Salamanca and 3% in Madrid) in recent years [31,32]. Infections with *D. immitis* in dogs range from 5% to 17% (Corsica, France), 10% to 34% (Greece), 3% to 14% (the Balkan Peninsula), and 1.52% to 46.2% (Turkey) [33,34]. On the southern shore of the Mediterranean basin, prevalence ranges from 4.7% to 14.5% (Tunisia), 1.4% to 24.5% (Algeria), and 12.1% to 16.1% (Morocco) [35-37]. Although heartworm infection has been reported in Egypt [13], no data are available about its prevalence. In Israel, no infection has been detected, and the only dog reported with heartworm infection in this country was imported from the United States [38].

Few data have been published on *D. repens* infections on dogs, perhaps because of its reduced virulence [29] and the absence of clinical signs in the majority of canine infections and the difficulty in diagnosing the infection [19]. The cases of autochthonous infection with *D. repens* have been reported in Portugal [39], Spain [40], Italy [41], France [42], Greece [43], Turkey [44], and the Balkan Peninsula [45]. On the other shore of the Mediterranean, native cases have been reported in Tunisia [35], Algeria [46], and Israel [47].

Regarding feline *D. immitis* and *D. repens* infections, they tend to be detected in the same areas as canine infections, but with a lower prevalence than those reported in dogs [32]. For example, recently published data have reported infections with *D. immitis* with a prevalence of 0.2% in Spain [32], 4.8-15% in Portugal [48-50], 0.3% in Italy [51], and 4.6% in Egypt [52]. Sporadic cases of *D. repens* infections in the cat have also been reported in some Mediterranean countries, such as Portugal and Italy [51,53].

Regarding human dirofilariosis in the Mediterranean basin, it appears that cases are reported mostly in areas where canine dirofilariasis exists, with an exception for some countries with reported human cases in which data regarding canine infection are unavailable and vice versa. Until 2000, most diagnosed cases were reported in the Mediterranean nations (Italy, France, Greece, and Spain), where *D. immitis* and *D. repens* are traditionally endemic [25]. Over the following decade, more cases were reported in other Mediterranean countries, including Turkey, Serbia, Croatia, Albania, Slovenia, and Tunisia [24]. In general, subcutaneous/ocular *D. repens* dirofilariasis predominates with more than 3500 human cases reported in Europe from 1977 to 2016 [19]. In Portugal, the first human case subcutaneous dirofilariasis (an imported case) was reported in 2015 [54]. In Spain, eight reported cases of subcutaneous infection have been reported in 2017 [55]. In France, several human cases due to *D. repens* have been reported, mainly in the south of the country and on the island of Corsica [56]. It is noteworthy that the majority of the cases of human dirofilariosis identified in Europe have been reported in Italy with an incidence that increases over time. For example, the number of published human cases in Italy increased from 4.5 per year from 1986-1998 to 15.6 per year from 1999-2009 [24].

### Diagnosis of infection

*Dirofilaria* infections in dogs and cats can be diagnosed in several ways. The detection of circulating microfilariae in the bloodstream of infected animals through concentration methods, such as the modified Knott test and filter test, is regarded as the gold standard [10]. Serological tests based on *D. immitis* antigen detection are also used in front line testing in cases of heartworm disease in dogs [25], but this method is not recommended in cats because of the low number of adult worms in the cardiorespiratory system, making it difficult to detect low concentrations of circulating antigens [57]. Other tools include thoracic radiography and echocardiography can be used especially in cats [58]. It is worth mentioning that polymerase chain reaction (PCR) and quantitative PCR are considered the most sensitive and accurate tools for detecting and discriminating microfilariae from different filarial worms because of concentration methods and morphometric analyses of blood microfilariae are not often reliable in terms of differentiating between species. This is due to the high morphological criteria similarity between certain filariae species, particularly in cases of mixed infections or in cases with low parasitemia [59].

Diagnosis of human dirofilariasis is based on subcutaneous nodules or an ocular localization of worms, through self-discovery of the infection by the patient. Pulmonary nodules are located deep within the body and are asymptomatic in a high percentage of cases, and only a fraction of lung nodules are accidentally identified during chest X-ray procedures, which are generally performed for reasons unrelated to dirofilariasis [25]. Finally, the molecular characterization of samples (tissue biopsies or filariae fragments) is essential to precisely identify the species because recent studies have reported that human subcutaneous and cutaneous-pulmonary dirofilariosis were due to *D. immitis* and *D. repens*, respectively [21,22], hence the need for molecular biology in the diagnosis of human dirofilariasis.

### Treatment and prevention of infection

The treatment of dirofilariosis due to *D. immitis* in dogs and cats is complex and frequently risky, due to the side effects of the massive destruction of worms in the bloodstream [25]. Therefore, before starting any treatment, it is necessary to choose an appropriate therapeutic strategy. For heartworm infection, the organic arsenical compound melarsomine hydrochloride (one injection of 2.5 mg/kg body weight followed at least 1 month later by two injections of the same dose 24 h apart) is the only effective drug available against adult *D. immitis* infections [10]. Nevertheless, the American Heartworm Society (AHS) [60] recommends the use of doxycycline and a macrocyclic lactone before the three-dose regimen of melarsomine for the treatment of heartworm disease in both sick and apparently healthy but infected dogs. Any method using macrocyclic lactones alone as a slow-kill adulticide is not recommended. Pulmonary thromboembolism is an inevitable consequence of successful adulticide therapy, but this complication can be reduced by restricting exercise during the recovery period (30-40 days) and by administering anti-inflammatory doses of glucocorticosteroids, such as prednisolone, which is routinely dosed at 0.5 mg/kg for the 1^st^ week and 0.5 mg kg once daily for the 2^nd^ week, followed by 0.5 mg/kg every other day for 1-2 weeks [60]. The AHS recommends the administration of doxycycline at 10 mg/kg twice daily for 4 weeks before the administration of melarsomine. In reality, doxycycline reduces *Wolbachia*, an endo-symbiotic bacterium of many filarial nematodes. These bacteria appear to play a major role not only in the metabolism and reproduction of the filariae but also in the host-parasite relationship by producing a transforming growth factor-beta-like activity inducing, inter alia, immunosuppression in the host [25]. Interestingly, certain macrolides have adulticidal properties [61]. Experimental studies have shown that ivermectin has partial adulticidal properties when used continuously for 16 months or more at preventive doses (6-12 mcg/kg/month) and >95% adulticidal efficacy if administered continuously for about 30 months [10].

Surgical intervention is advised when several worms have been displaced into the right cardiac chambers, producing the sudden onset of caval syndrome (dirofilarial hemoglobinuria) [25]. Adulticide therapy is recommended a few weeks post-surgery to eliminate all remaining worms [60]. Concerning adulticidal therapy of canine and feline *D. repens* infection, there is no consensus on a treatment with a marketing authorization. The usual treatment protocol combines a subcutaneous injection of the macrocyclic lactone with prednisolone and doxycycline for 1 month. A novel approach for the treatment of cardiopulmonary dirofilariosis is targeting the *Wolbachia* rickettsial endosymbionts (i.e., the bacteria are essential for the filarial worms’ survival). Treatment with tetracyclines has been reported to damage *D. immitis*, even causing death of adult worms, presumably by elimination of *Wolbachia* from the worm [62]. In general, the protocols for the treatment of dogs infected with *D. immitis* associating ivermectin (conventional antiparasitic treatments) and doxycycline. Only a few authors describe the use of this combination, in particular, implementation of the following protocol on two cases of natural infestation: Doxycycline 10 mg kg day for 30 days and ivermectin 6 mg/kg every 15 days for 6 months [63]. Recommended surgical interventions include either the removal of nodules or the removal of adult worms from the nodule [25].

For the treatment of human dirofilariasis, patients often receive antiparasitic treatment, including ivermectin and albendazole as well as a microfilaricidal agent such as diethylcarbamazine in the rare cases of microfilaremia. However, there is no strong support for these treatments [25,64]. Surgical removal of the worm remains the treatment of choice, in most cases due to the suspicion of a malignant origin of the nodule or the presence of worms in ocular locations [25]. Removal of subcutaneous nodules or worms from the ocular conjunctiva is a simple procedure, but surgical intervention is much more complex for pulmonary, ocular, retro-ocular, or other internal locations [25].

As for control strategies for dog and cat dirofilariosis, to control pathogenic and/or zoonotic parasites, AHS recommends year-round administration of chemoprophylactic drugs [60]. Monthly administration of topical or oral macrocyclic lactones throughout the transmission season is effective against *D. immitis* third-stage larvae (L3) and L4, which has developed in the previous days and thus prevent disease caused by adult worms. Several approved compounds of macrocyclic lactones (e.g., ivermectin, milbemycin oxime, moxidectin, and selamectin) administrated alone or in combination with other parasiticides are available for oral administration or topical application [3]. Finally, external repellent antiparasitic agents containing synthetic pyrethroids, deltamethrin, or permethrin are particularly suitable for the control of vectors of dirofilariosis (a repellent effect against mosquitoes) and their efficacy has been demonstrated both experimentally and in field studies [65-67]. One of them, based on a dinotefuran-permethrin-pyriproxyfen combination has recently demonstrated its effectiveness in the prevention of canine dirofilariosis in combination with a macrocyclic lactone [68].

## T. callipaeda Infection

### Etiology

Thelaziosis due to the *T. callipaeda* (*Spirurida* and *Thelazidae*) eyeworm is a parasitic nematode transmitted by *Phortica variegata* (*Diptera*, *Drosophilidae*, and *Steganinae*), a drosophilid that feeds on lachrymal secretions of mammals [69]. This worm was described for the first time by Railliet and Henry [70] for specimens collected from the nictitating membrane of a dog in Rawal Pindi, Punjab, India. *T. callipaeda* is considered to be an emerging pathogen in Europe, and several cases have been reported in dogs, cats, and wild carnivores in areas where the disease did not previously exist [71]. In humans, two species have been implicated in the infection: *T. callipaeda* and *T. californiensis*. However, only *T. callipaeda* infection has been increasingly reported in humans in Europe following the first report from Northern Italy [72]. In addition, the first report in dogs came from the same area [73]. This explains why Italian researchers were the first to study this new helminthosis in the Mediterranean region. In their studies, Otranto *et al*. [74] reported the presence of *T. callipaeda* and the prevalence of the infection in dogs, cats, and foxes [75]. In definitive hosts, adult and larval stages of *T. callipaeda* are responsible for an eye disease with mild (e.g., conjunctivitis, epiphora, and ocular discharge) to severe (e.g., keratitis, and corneal ulcers) clinical signs [69].

### Host and life cycle

Kozlov [76] reported on the life cycle of this worm. Dogs, cats, beech martens, foxes, wolves, rabbits, hares, and humans are the usual definitive hosts for *T. callipaeda*. Female adult worms produce a large number of first-stage larvae (L1) that are deposited in the lachrymal secretions of the hosts and ingested by secretophagous dipteran insects. The first-stage larvae (L1) are ingested by the intermediate host (*P. variegata*). After 3 weeks and two molts, the larvae become infective third-stage larvae (L3) and migrate to the fly’s mouthparts, where they remain until the fly feeds on the tears of the definitive host. The L3 of *Thelazia* feeds on the lachrymal secretions from infected hosts and develops into the adult stage in the conjunctival sac and peribulbar tear film within about 35 days [74].

The life cycle of *T. callipaeda* was studied in naturally infected dogs by Otranto *et al*. in 2003 [74]. The existence of a seasonal periodicity in the reproductive cycle of female *T. callipaeda*, coinciding with the presence/absence of the vectors, has been demonstrated. Their results showed that the first-stage larvae were found in the lachrymal secretions of dogs in the summer, ready to be ingested by flies feeding around the eyes [74].

### Epidemiology

*T. callipaeda* infection has long been considered to be present only in Russia and the Far East, including Indonesia, Thailand, China, Korea, India, Japan, Myanmar, and Burma [77]. In the Mediterranean basin, Italy is considered as a historical focus for *T. callipaeda* because this parasite was reported for the 1^st^ time in 1989 in dogs living in the Piedmont region of the country [73]. Subsequently, prevalence studies were also conducted in dogs in the northern and southern regions, and the highest prevalence was reported in the south of the country (up to 60% of the canine population) [74]. In the same area, cats have been also found to be infected with *T. callipaeda* [74]. Canine and feline thelaziosis was also detected in France in 2006 [78]. Recent investigations conducted in Southwestern France showed that canine thelaziosis is more prevalent than previously thought because in 2016, in two veterinary clinics located in the department of the Dordogne, 119 cases were diagnosed positive for *T. callipaeda*. According to the same study, in two other clinics located in the Landes department, more than 60 cases were reported in 2016 [79]. The first case of canine thelaziosis was observed in La Vera region, central western Spain, in 2010 [80]. In 2011 and 2017, two studies showed that the prevalence of canine thelaziosis in the same region (La Vera) increased exponentially to reach 40% and 68%, respectively [81,82]. The first case of canine thelaziosis was reported in the North of Portugal in 2012 [80]. In Portugal, an autochthonous case of feline thelaziosis was reported in 2013 [83]. In the same year, *T. callipaeda* infection was reported for the first time in foxes (*Vulpes vulpes*) [84]. The first indigenous case of ocular thelaziosis in a dog from Greece was reported in 2015 [85]. Meanwhile, a recent study showed that from 2014 to 2016, a total of 46 dogs and three cats tested positive for *T. callipaeda* infection in different areas of the northern and central Greece [86]. The disease has also been diagnosed in dogs (n=51) and cats (n=2) in Balkan countries, including Croatia, Bosnia and Herzegovina, and Serbia. Interestingly, in Bosnia between 2011 and 2014, thelaziosis due to *T. callipaeda* was observed in 51/184 (27.71%) of red foxes [87]. As far as the other Mediterranean countries are concerned, no cases have been reported to date.

As for human thelaziasis, the first indigenous cases of this zoonotic disease were reported in Italy and France in 2005 [88] and in Spain in 2012 [89]. Recently, two human cases have been reported in Serbia [90] and in Croatia [91]. All these cases of human thelaziasis appear to have been reported during the summer months (June-August), which is the period of *T. callipaeda* vector activity (late spring to fall in Southern Europe) [92]. To the best of our knowledge, no human cases have been reported in other Mediterranean countries.

*P. variegata* is the only confirmed vector of *T. callipaeda* in the Mediterranean countries where the disease has been reported [74]. A study conducted byOtranto *et al*. [93] in an area of Italy with a high prevalence of canine thelaziosis, reported that in a total of 969 *P. variegata* collected between April and November of the same year, the number of specimens collected weekly was related to climatic and environmental parameters (e.g., temperature, relative humidity, and total rainfall) recorded daily at the collection site. The largest number of *P. variegata* was collected in July and August (507/969). According to these authors, the ecological niche model predicted that large areas of Europe (obviously all the Mediterranean countries) are likely to represent suitable habitat for this species of drosophilid [93]. In their study, Marino *et al*. [82] reported for the first time in Spain the implication of *P. variegata* as a potential vector of *T. callipaeda* with an infection rate of 7.5% (28/371).

### Diagnosis of infection

**Figure-4:**
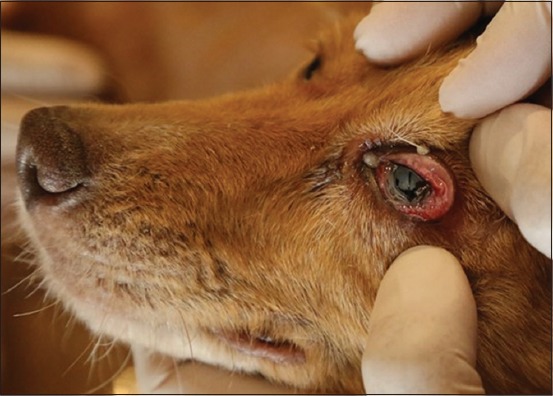
Conjunctivitis in a dog with *Thelazia callipaeda*.

In canine and human thelaziosis, both adult and larval stages of nematodes live under the nictitating membrane of the eye [74]. Thus, the diagnosis in animals and humans is usually based on finding the adult and/or larval eyeworms mostly in the conjunctival sac or medial or lateral canthus of the eye (Figure-4) following a clinical and ophthalmological examination of infected animals or humans [94,95].

The specific identification of the parasite, based on the morphological identification criteria, is central to the diagnosis of thelaziosis [95]. Briefly, adult females of *T. callipaeda* measure 12-18.5 mm in length and 370-510 μm in width and are characterized by the position of the vulva located anterior to the esophageal-intestinal junction. Adult males measure 7.7-12.8 mm in length and 338-428 μm in width in the midportion of the body and present five pairs of post-cloacal papillae [95]. In addition to detecting adult nematodes, the typical newborn L1 can be seen microscopically in eye secretions [94].

### Treatment and prevention of infection

The adults and larvae of *T. callipaeda* can be mechanically removed by rinsing the conjunctival sac with sterile physiological saline after local anesthesia with 1% dicaine [94]. As for the treatment of infected domestic animals, it has been demonstrated that the topical instillation of organophosphates or moxidectin 1% is effective against *T. callipaeda* [96]. Some ectoparasiticide products, such as imidacloprid (10%) and moxidectin (2.5%) spot-on formula, appear to be effective in terms of controlling dog thelaziosis within 5 (90.47%)-9 (95.23%) days of treatment [95]. In addition, in a field study conducted by Lechat *et al*. [97] showed that the monthly application of a spot-on formula containing 10% imidacloprid and 2.5% moxidectin was highly effective in preventing *T. callipaeda* infection in a population of dogs living in an endemic area in France. In another study, the efficacy of the prophylactic use of monthly treatment with milbemycin oxime showed 90% efficacy in reducing *T. callipaeda* infection rate in dogs [98]. Chemoprophylaxis of dogs against *T. callipaeda* infection is essential to reduce its prevalence and the risk of transmission of this eyeworm to humans, as dogs may act as reservoirs for human infection.

## *O. lupi* Infection

### Etiology

*O. lupi* (*Spirurida* and *Onchocercidae*) is a nematode parasite causing ocular disease (i.e., conjunctivitis, ocular swelling, photophobia, lacrimation, discharge, and exophthalmia) in canines and felines [99]. The parasite was first described in 1967 in the periocular tissues of a Caucasian wolf (*Canis lupus*) from Georgia. After confirming the first human infection in 2011 in Turkey [12], *O. lupi* was recognized as an emerging zoonotic pathogen which has been incriminated as the etiological agent of several human cases in the United States and Old World countries in Europe, Africa, and the Middle East [100-102]. Black flies (*Diptera* and *Simuliidae*) *Simulium tribulatum* are reported as being a putative vector of this nematode [11].

### Hosts and life cycle

The life cycle of *Onchocerca* spp. consists of two phases, one in black flies (vectors–intermediate hosts) and the other in definitive hosts (humans and animals). Nevertheless, the *O. lupi* life cycle, including the vector and its primary reservoir host, remains unknown [99]. As in several other species of *Onchocerca*, black flies may play a role in the transmission of *O. lupi* in dogs and humans, but this hypothesis remains to be confirmed with experimental studies [103].

### Epidemiology

Canine ocular onchocercosis has been reported worldwide. In Southern Europe, the number of detected cases caused by *O. lupi* appears to be increasing. For example, in a study conducted in Greece and Portugal, out of a total of 107 dogs, 9 (8%) were skin snip-positive for the parasite [104]. Recently, the infection was reported in Spain, with a prevalence of 4.8% (5/104) of exanimated dogs [105]. For the first time in the Mediterranean Basin, cat onchocercosis was reported in 2015 in Portugal [106].

As for human ocular onchocerciasis, it is important to note that the zoonotic role of *O. lupi* was first highlighted in 2002 in two suspected human cases in Albania and Russia [107]. However, it was only recently, from 2011 onward, that human cases have been confirmed and described in Turkey and Tunisia [12,102,108].

### Diagnosis of infection

In the majority of cases, canine onchocercosis was reported as an acute or chronic ocular disease. In acute cases, conjunctivitis, exophthalmos, periorbital swelling, photophobia, discomfort, lacrimation, and discharge have been observed without granuloma or cyst formation around the worms [109]. In chronic cases, the clinical signs are variable (e.g., exophthalmos, conjunctival congestion, discharge, periorbital swelling, granuloma formation, and lacrimation).

Conventionally, the diagnosis of canine onchocercosis consists of the detection of dermal microfilariae from the skin and adult worms found in surgically removed tissues (e.g., nodules) or at necropsy followed by their morphological and/or molecular identification [3,99]. Dermal microfilariae of *O. lupi* may be detected in sediments of skin samples or skin biopsies soaked in a saline solution for a few minutes at 37°C and stained with methylene blue and then identified using morphological criteria [110]. Briefly, dermal microfilariae are characterized by an unsheathed body 98-118 µm long by 5-7 µm wide, with rounded anterior extremity bearing a tiny tooth and a bent tail, 11.7 µm long [110]. However, this technique is considered an invasive procedure and requires considerable technical skills to isolate and identify parasites [3]. Recently, a commercially ELISA test based on the detection of parasite antigens in canine sera has been developed [111]. However, the sensitivity and specificity of these tests remain a subject of discussion. Molecular tools such as PCR and DNA sequencing have also been proposed for the identification of microfilariae and adult stages of *O. lupi* [112].

### Treatment and prevention of infection

Treatment consists of surgical interventions and chemotherapy together with topical medical treatment of the ocular signs. In dogs and cats, surgical excision of as many periocular nodules and cysts as possible was found to be a suitable treatment option [109]. For chemotherapy of canine ocular, anthelminthic drugs, e.g., the microfilaricide ivermectin and the adulticide melarsomine associated with antibiotics, were used [101]. Systemic and topical steroids were successfully applied to avoid the periorbital pruritic inflammation usually noticed 2-3 days after adulticide melarsomine treatment, as well as for the control of uveitis and orbital disease [113].

For human ocular onchocercosis, surgical excision has led to a complete cure. Exceptionally, additional anthelminthic drugs such as ivermectin (150 µg/kg/dose, as a single dose to be repeated every 3 months for 5 years) and/or albendazole (15 mg/kg/day for a total of 12 days) have been administered [114,115]. In reality, there is no consensus on the treatment of *O. lupi*. However, because ivermectin is known to kill the larvae of *O. volvulus* and accelerates the death of adult worms [116], this ivermectin-based protocol was applied against *O. lupi* infection. Finally, corticoid drugs such as dexamethasone may be administered after surgery and cryotherapy because of generalized urticaria [117].

There are no effective control methods, but insect repellents can reduce vector attacks. The role of ivermectin, milbemycin, and other heartworm preventive medications commonly used in dogs and cats is unknown. These medications would probably kill microfilariae, but their efficacy against infective L3 larvae of *O. lupi* is unknown. Komnenou and Koutinas (2007) [113] suggest that the monthly administration of milbemycin for 6 months (from April to October, coinciding with peak black fly and gnat activity) could prevent canine onchocercosis. All these medications may play a role in preventing infection in pets or in preventing infected pets from being used as reservoir hosts, thereby reducing the transmission of this infection [101].

## *Acanthocheilonema* Infection

### Etiology

*A. reconditum* (Grassi 1889) and *A*. *dracunculoides* (Cobbold, 1870) are the causative agents of the canine subcutaneous filariosis due to subcutaneous nodule formations, and a largely neglected and poorly known species of filariae [14,118]. Its infestation in carnivores, the only definitive hosts, is not clinically important, although they may manifest an elevated eosinophil and leukocyte count [14]. *A. reconditum* microfilariae averages about 280 µm in length and 4.7-5.8 µm in width with a round curved body, a prominent cephalic hook, a blunt anterior end, and a filiform posterior end. Adult males and females average 13 mm and 17-32 mm in length, respectively [119]. *A. dracunculoides* microfilariae average about 287 µm in length and 10 µm in width with a sharp and extended cephalic hook, a conical anterior end, and a straight posterior end. Males are 15–32 mm long and 0.1–0.2 mm wide, while females are 30-60 mm long and 0.1-0.3 mm wide [120].

### Host and life cycle

*Acanthocheilonema* has an indirect life cycle with the development of infective larvae that are carried by fleas (genus *Ctenocephalides*, *Pulex*, and *Echidnophaga*), ticks (*Rhipicephalus sanguineus*) and lice (genus *Linognathus*, and *Heterodoxus*) for *A. reconditum*, and by lice (*Hippobosca longipennis*) and ticks (*R. sanguineus*) for *A*. *dracunculoides* [119,121,122]. Adult worms of *A. reconditum* occur in the subcutaneous tissues and, exceptionally, in other common sites of infestation, including body cavities and the kidneys [14]. The predilection site for adult male and female *A*. *dracunculoides* is mainly the peritoneal cavity [121]. Adult females produce L1-stage larva, which circulates in the peripheral blood of the definitive host. Microfilariae ingested during blood feeding develop into infective L3-stages and eventually accumulate in the mouthparts of the respective arthropods involved. Dogs are infected when bitten by infected hematophagous arthropods [119].

### Epidemiology

*A. reconditum* infection has a worldwide distribution, including in the United States, South America, Oceania, and many African and European countries [14]. *A*. *dracunculoides* occurs in Europe, Asia, and Africa [118,122]. Regarding Southern Europe, *A. reconditum* has been reported in dogs in Greece [43], Spain [123], France [124], and Italy [125,126], with a prevalence ranging from 0.5% to 15.9%. As for other Mediterranean countries, no canine cases due to *A. reconditum* have been reported. Moreover, *A. reconditum* infection in wild carnivores (such as *V. vulpes*) has been diagnosed in Italy, at least in two epidemiological studies, with a prevalence of 10.89-9.09% [127,128]. Finally, this parasite (*A. reconditum*) has occasionally been reported as a zoonotic agent, especially after humanist first discovery in a human eye [14], but to the best our knowledge, no human cases have been reported in the Mediterranean basin. Dogs infected with *A*. *dracunculoides* have been identified in Portugal [26], Spain [123], Italy [129], Algeria [130], Morocco [131], and Tunisia [132]. The prevalence reported in these countries ranges between 2.7% and 13.19%. No cases have been reported in the other countries of the Mediterranean region. Nevertheless, *A. dracunculoides* infection has been reported in red fox (*V. vulpes*) in Italy [128]. As for human infection with *A. dracunculoides*, no cases have been reported in the Mediterranean countries.

### Diagnosis of infection

Microfilariae for both species are hematic-circulating and can be detected in blood samples. The diagnostic methods for *Acanthocheilonema* infections, therefore, include morphological observation of circulating microfilariae by stained blood smears, direct wet smears, and modified Knott’s technique, as well as through the isolation of adult worms followed by a morphological identification [133,134]. Histochemical or immunohistochemical staining of circulating microfilariae has also been performed [134,135]. Finally, molecular diagnostic approaches are also increasingly used for species identification, research, and monitoring purposes [112].

### Treatment and prevention of infection

It should be noted that no molecules are marketed to prevent *Acanthocheilonema* infection, perhaps due to their low pathogenic impact. Traversa *et al*. [136] suggest that the spot-on Advocate® (moxidectin and imidacloprid) is of interest in the prevention of the infestation by *A. reconditum*: On the one hand, by avoiding infestation by the vectors of these parasites and, on the other hand, by eliminating the infesting larvae. In fact, moxidectin spot-on has already succeeded in eliminating *D. repens* circulating microfilariae in naturally infected dogs [137].

## *Cercopithifilaria* Infection

### Etiology

The genus *Cercopithifilaria* comprises up to 28 species that parasite a broad range of carnivores and ungulates [16,138]. However, in the Mediterranean region, *Cercopithifilaria* described in dogs includes at least three species: *Cercopithifilaria grassii*, *Cercopithifilaria bainae*, and *Cercopithifilaria* spp *. sensu* Otranto *et al*. 2013 [139]. In Europe, these three species have been morphologically and molecularly characterized [16,139]. Adult *Cercopithifilaria* spp. usually dwell beneath the cutaneous tissues of infected dogs [138], while their larvae are distributed unevenly in the superficial dermal tissues, mostly present on the skin of the head, ears, and neck [140]. *Cercopithifilaria* spp. found in dogs are less virulent compared to other filaroids, and only a few clinical and histological alterations have been recorded in the course of infestation [140].

The dermal microfilariae of *C. grassii* are 567-660 μm long, 12.2-15.5 μm wide, and with blunt anterior extremity. *C. bainae* is 185.2 μm long by 6.6 μm wide with a blunt anterior end. Microfilariae *Cercopithifilaria* spp. are 182-190 μm long by 8.5-11 μm wide with a blunt anterior end [17].

### Host and life cycle

The life cycle involves infective larvae transmitted by the brown dog tick *R. sanguineus*, which is also considered to be an intermediate host of this nematode [141]. In reality, infective larvae (L3) reside in the dog’s dermis, where they mature to adults, producing ovoviviparous microfilariae (L1). During a blood meal, ticks ingest microfilariae which in turn develop into infective larval stages (L3), within approximately 30 days [141]. Dogs are infected when bitten by infected hematophagous arthropods.

### Epidemiology

In 1907, Noè described for the first time a nematode with dermal microfilariae in dogs as *Filaria grassii* (syn. *C. grassii*). In 2011, Otranto *et al*. [16] reported in a dog from Sicily (Italy) a *Cercopithifilaria* spp. *sensu* Otranto *et al*. [16] (*Cercopithifilaria* sp.), which was morphologically different from *C. grassii*. Later, this filaroid (*Cercopithifilaria* spp.) was detected in dogs in Spain, Greece, and different Southern Italian regions (including Basilicata and Sicily), with a prevalence ranging from 10.5% to 15% according to the technique used (e.g., microscopy of skin sediments or PCR on skin samples) for the detection [7,142]. A recent study conducted in dogs (n=102) in the Algarve region of Portugal reported for the first time the occurrence of *Cercopithifilaria* spp. with the presence of the three known species: *C. bainae* (9.8%), *C. grassii* (3.9%), and *Cercopithifilaria* spp. *II sensu* Otranto *et al.*, 2013 (13.7%) [143]. Concerning other Mediterranean countries, no cases have been reported.

### Diagnosis of infection

The diagnosis of the infection is based on the detection and morphological identification of the dermal microfilariae, which is usually performed by examining skin snip samples soaked in saline solution to recover microfilariae [17]. Hence, these parasites are largely unknown to the scientific community, and their relevance in veterinary medicine is considered minimal.

### Treatment and prevention of infection

No treatment is available against the parasites of the genus *Cercopithifilaria*. Nevertheless, an infestation indicates that the dog has been exposed to the bites of *R. sanguineus* ticks, a vector of *Cercopithifilaria* spp. It should be noted that in the case where *C. bainae* microfilariae were found in the synovial fluid of a dog with chronic polyarthritis, repeated treatments with milbemycin oxime did not result in clinical improvement [144]. The best way to protect dogs against cercopithi filariosis is to protect them against tick infestation by regularly using external antiparasitic drugs. This avoids tick bites (*R. sanguineus*) and, consequently, the transmission of nematode parasites.

## Conclusion

Through this review on VBNDs of companion animals and humans in the Mediterranean region, it is clear that dirofilariosis due to both *D. immiti* s and *D. repen* s species is the most important VBND due to two reasons: (i) many animal and human cases have not only been reported in recent years in historically endemic countries (Italy, France, Portugal, and Spain) but also in non-traditionally endemic areas (Algeria, Tunisia, and Morocco) and (ii) the invasion of the Mediterranean region with tiger mosquitoes (*Aedes albopictu* s), a species that has been proven to be a competent vector for *D. immiti* s and *D. repens*. This further increases the risk of the re-emergence of animal and human dirofilariasis in the Mediterranean Basin.

*O. lupi* and *T. callipaeda* filarioids are also increasingly important in the Mediterranean region because not only canine cases have been reported, increasingly in some areas, but also their zoonotic aspect has been noted, particularly for *O. lupi*, with human ocular infections being reported in Turkey and Tunisia, in 2001 and 2012, respectively. The significance of the *T. callipaeda* infection is due to the natural vector “*P. variegate*,” which appears to be present in numerous European countries situated around the Mediterranean.

Finally, *Cercopithifilaria* spp. and *Acanthocheilonema* spp. infections in dogs appear to be less important compared to other VBNDs because these parasites (except for *A. reconditum*) have not been reported in humans. It is important to note that much higher incidences of *Cercopithifilaria* spp. infection have been reported in Italy and Greece. This can be explained by the interest the researchers paid to this filarioid infection in these countries.

The current epidemiologic patterns in endemic regions and in previously infection-free areas show that they are spreading [136,145]. These diseases are rarely controlled by a single approach. The best way of controlling any one of them is likely to be a combination of vector, pathogen, and reservoir controls and then only be taking an integrated approach.

## Authors’ Contributions

DT designed and prepared the manuscript as a part of his research. BD and PP carried out proofreading and made critical comments in this manuscript. All authors read and approved the final manuscript.
